# Exploring COVID-19 Daily Records of Diagnosed Cases and Fatalities Based on Simple Nonparametric Methods

**DOI:** 10.3390/idr13020031

**Published:** 2021-04-01

**Authors:** Hans H. Diebner, Nina Timmesfeld

**Affiliations:** Biometry and Epidemiology, Department of Medical Informatics, Ruhr-Universität Bochum, 44780 Bochum, Germany; nina.timmesfeld@rub.de

**Keywords:** SARS-CoV-2, COVID-19 pandemic, nonparametric methods, case–fatality ratio

## Abstract

Containment strategies to combat epidemics such as SARS-CoV-2/COVID-19 require the availability of epidemiological parameters, e.g., the effective reproduction number. Parametric models such as the commonly used susceptible-infected-removed (SIR) compartment models fitted to observed incidence time series have limitations due to the time-dependency of the parameters. Furthermore, fatalities are delayed with respect to the counts of new cases, and the reproduction cycle leads to periodic patterns in incidence time series. Therefore, based on comprehensible nonparametric methods including time-delay correlation analyses, estimates of crucial parameters that characterise the COVID-19 pandemic with a focus on the German epidemic are presented using publicly available time-series data on prevalence and fatalities. The estimates for Germany are compared with the results for seven other countries (France, Italy, the United States of America, the United Kingdom, Spain, Switzerland, and Brazil). The duration from diagnosis to death resulting from delay-time correlations turns out to be 13 days with high accuracy for Germany and Switzerland. For the other countries, the time-to-death durations have wider confidence intervals. With respect to the German data, the two time series of new cases and fatalities exhibit a strong coherence. Based on the time lag between diagnoses and deaths, properly delayed asymptotic as well as instantaneous fatality–case ratios are calculated. The temporal median of the instantaneous fatality–case ratio with time lag of 13 days between cases and deaths for Germany turns out to be 0.02. Time courses of asymptotic fatality–case ratios are presented for other countries, which substantially differ during the first half of the pandemic but converge to a narrow range with standard deviation 0.0057 and mean 0.024. Similar results are obtained from comparing time courses of instantaneous fatality–case ratios with optimal delay for the 8 exemplarily chosen countries. The basic reproduction number, $R_{0}$, for Germany is estimated to be between 2.4 and 3.4 depending on the generation time, which is estimated based on a delay autocorrelation analysis. Resonances at about 4 days and 7 days are observed, partially attributable to weekly periodicity of sampling. The instantaneous (time-dependent) reproduction number is estimated from the incident (counts of new) cases, thus allowing us to infer the temporal behaviour of the reproduction number during the epidemic course. The time course of the reproduction number turns out to be consistent with the time-dependent per capita growth.

## 1. Introduction

The current (2020/2021) hard-to-tackle flood of publications on virological, epidemiological, and sociological aspects of the SARS-CoV-2 coronavirus and its related disease COVID-19 [1,2] along with the concurrent demand by many public health institutions and authorities for intensifying corresponding research in order to quickly gain a deeper understanding of the pandemic entail a dilemma for researchers. On the one hand, due to the inevitable lack of overview on existing publications, it is almost impossible to ensure that newly published work does not merely add redundancy, thus amplifying the flood. On the other hand, hesitating to submit may prevent quality research from being published.

In spite of this dilemma, the present paper is motivated by the hope that the simplicity of the proposed mathematical methodology applied to data on the incidence of COVID-19 cases leads to meaningful insights. Moreover, it can be generalised and transferred to other epidemics beyond SARS-CoV-2/COVID-19. Therefore, we largely follow the appraisal by S. Jahedi and J. York [3] that complex models, such as dynamical multi-compartment models, are unlikely to be understood by nonexperts. Moreover, complex models are usually parametric in nature and constructed in order to eventually supply estimates of the involved parameters, such as the basic reproduction number $R_{0}$ or the instantaneous effective reproduction number R(t). However, most of these parameters, such as R(t), are largely time-dependent and are contingent on changing public health policies and social behaviour. Modelling then relies on debatable assumptions on impact and timing of these “soft” criteria and a priori guesses of some parameter values.

Arguably, the basic reproduction number, $R_{0}$, and the effective reproduction number, R(t), are the most important key figures that classify an epidemic [4]. Originally derived in the context of demographic structured population modelling, where $R_{0}$ is defined as the moment of order zero (hence the subscript 0) of the net maternity function, its definition had to be adapted within the scope of infection epidemiology [4,5]. In the latter context, $R_{0}$ is defined for a fully susceptible population at the beginning of an epidemic and refers to the number of secondary cases caused by an index case, whereas R(t) refers to the usually time-dependent analogue during the course of the epidemic when the population is no longer fully susceptible. We learn from [6] that Germany’s “patient zero” caused three (detected) secondary infections in Germany and a further (detected) secondary case after returning to China. However, the strict epidemiological definition of $R_{0}$, being an integer number, is of limited practical use. Therefore, the “index case” has to be conceived as an “average infectious individual” of the given population, i.e., $R_{0}$ can be derived as the expectation value of a Poisson distribution of the number of secondary cases, which emphasises the idea of a “representative” case.

Using only secondary infections caused by the index case to estimate $R_{0}$ necessitates knowledge about the generation time distribution of the infection, thus creating another insufficiency. In practice, there exist a number of different methods that aim to estimate $R_{0}$ from the early approximately exponential phase of an epidemic (see, e.g., [4,7]). An alternative approach to estimate $R_{0}$ is related to parameter estimations from fitting differential equation-based epidemiological models to incidence data [8], if available.

Most of the freely available data files contain daily counts of newly diagnosed COVID-19 cases as well as recorded deaths, such as the database maintained by the Johns Hopkins University [9,10]. Therefore, a proportion of usually more or less symptom-free infectees remains undetected and does, therefore, not appear in the dataset. Some datasets (e.g., [9,11,12]) additionally contain time series of the number of recovered patients; however, these records usually do not result from rigorously confirmed serological diagnoses but rather from applications of elsewhere-estimated average recovery times. Thus, only the records of diagnosed cases and deaths are by and large reliable, at least for most of the countries with an efficient health care system. For a few countries, the reliability of COVID-19 reports and recordings might be questionable. With respect to Germany, substantial delays in reporting fatalities have been criticised [13] and hampers reliable analyses.

Occasionally, historical data have later been revised by some countries (cf. annotations in [11]), which may lead to inconsistencies upon reproducing the analysis. However, there are some more serious problems that complicate analysis. As mentioned above, infected individuals with mild or no symptoms are usually not detected. Coverage and frequency of testing heavily depends on the local policy as well as on the availability and accuracy of diagnostic equipment (cf. [14,15,16]) and may change in the course of time, leading to a varying ratio of reported counts of cases to unreported numbers of infections. Thus, the recorded diagnosed COVID-19 cases are likely a temporarily nonconstant proportion of the number of actual infections. Projected scenarios and forecasting from sophisticated differential equation-based epidemiological models (e.g., SIR [17], SEIR [18], fractional SIR models [19], and other [20]) are based on debatable assumptions concerning the policy-dependent and, hence, time-dependent amount of unreported cases and parameters that are crucially contingent on contact frequencies, hygiene. and a plethora of other social conditions [21,22]. Eventually, estimating nonconstant parameters directly from fitting SIR models may no longer be feasible such that model-independent (nonparametric) methods for parameter estimations are required. That way, the estimated time courses of parameters can either be fed into dynamical modelling or autonomously used to draw inferences.

The approach presented here refrains from discussing complex parametric models and avoids doubtful assumptions on the impact of policies. It goes without saying that large-scale cross-sectional epidemiological studies (for paradigmatic small-scale studies, see [16,23]) are needed to obtain a reliable quantification of all relevant parameters required for a significant assessment of the pandemic. Meanwhile, the present work provides a comprehensible nonparametric exploration of the existing records of counts of diagnosed cases and fatality events. In brief, we reveal some interesting hallmarks and estimate crucial parameters of the COVID-19 pandemic from “naked” incidence data without making questionable assumptions that are not directly supported by the dataset. To be specific, we supply country-specific estimates of fatality–case ratios (often confusingly denoted as case–fatality rates) as well as estimates of the average duration from diagnosis to death. In addition, the value of the reproduction number is estimated based on two different types of approximations. Alternatively, the per capita growth rate (Malthus parameter) of new cases reflects the reproduction process without knowledge of the generation time. The presented analysis is exemplarily applied to a few countries (Germany, France, Italy, Spain, Switzerland, the UK, the USA, and Brazil), which are compared to the worldwide situation. Some details are highlighted for Germany. Due to its manageable complexity, the proposed calculations are easily portable not only to datasets of other countries involved in the COVID-19 pandemic but also generally to other epidemic incidence data.

## 2. Methods

### 2.1. Observational Data

In this work, data on the geographic distribution of COVID-19 cases worldwide are used, which are freely distributed online by the Johns Hopkins University (JHU, cf. [9]) and made available in a computer readable format by [10]. Of note, in a preprint version of this article [24], we also used data provided by the European Centre for Disease Prevention and Control (ECDC) [11]; however, ECDC stopped providing data sampled on a daily bases starting in mid-December 2020 and switched to weekly updates instead.

The data file contains daily counts of newly diagnosed COVID-19 cases and deaths, stratified by country. The last evaluation date used in this work is 28 January 2020. The JHU dataset also contains population size information for each country (2019 census), which we use for normalisations (per capita incidence). The world population size was assumed to be 7.8e9. In [24], incidence time-series data for the German epidemic provided by the German Robert–Koch Institute (RKI) [12] were used to contrast the results, where it appeared appropriate. With respect to reporting date, the differences between JHU data and RKI data are completely insignificant. With respect to the following analysis, we have to keep in mind that delays in reporting cases and fatalities can exceed four weeks [13]. Therefore and due to confusing algorithms behind corrections of reporting dates of confirmed cases for symptom onset offered by the RKI, here, we skip to updating the comparison with RKI data. Throughout the article, the results from the analysis refer to the JHU data.

### 2.2. Mathematical and Statistical Modelling

#### 2.2.1. Asymptotic and Instantaneous Fatality–Case Ratios

Time within the dataset refers to calendar time with a one per day (1/d) sampling frequency. Therefore, in the following, *t* refers to a discrete time variable with a spacing of 1d. To simplify the mathematical notation, t=0 refers to the date of first observation and subsequent time points are denoted as t=0,1,⋯,T, with t=T being the current or final observation time. However, for an intuitive comprehension of the time scales, the time-axis labels of plots are given in the calendar date. The number of newly diagnosed cases at date (time point) *t* are denoted as cases(t), whereas the cumulative sum of cases up to date *t* is denoted as cumCases(t) with

(1) $$cumCases\left(t\right)=\sum_{i=0}^{t}cases\left(i\right).$$

Analogously, the number of daily newly recorded fatalities at date *t* is denoted as deaths(t) and the total number of registered deaths up to time point *t* is denoted by cumDeaths(t).

Since the notion of a “rate” is occasionally used in an ambiguous way in publications, particularly with respect to “death rate”, here, we explicitly introduce definitions of the fatality measures that are applied to the COVID-19 data:

1. Delay−Δt asymptotic fatality–case ratio:
  (2) $$AFCR_{\Delta t}\left(t\right)=\frac{cumDeaths(t)}{cumCases(t-\Delta t)}\;\forall \;t\geq \Delta t$$
2. Delay−Δt instantaneous fatality–case ratio:
  (3) $$IFCR_{\Delta t}\left(t\right)=\frac{deaths(t)}{cases(t-\Delta t)}\;\forall \;t\geq \Delta t.$$

The delay time Δt represents a shift between the two time series cases(t) and deaths(t). Choosing Δt to be the mean duration from diagnosis to death is expected to yield the most reliable fatality–case ratio. Confer the following section for a proper optimisation procedure. At the end of the pandemic, formally for t→∞, $AFCR_{\Delta t}\left(t\to \infty \right)$ becomes independent of Δt and converges, at least in the ideal case, to a value that corresponds to what is frequently called the case–fatality rate. In real-life applications (e.g., in cross-sectional studies such as [16]), case–fatality rates are often estimated before the epidemics come to a halt and represent, therefore, only interim values $AFCR_{0}\left(t\right)$ at time *t* using delay Δt=0. The choice of Δt=0 can lead to misleading results when the case–fatality rate is estimated at an early stage of the epidemic due to the likely finite survival time Δt>0. An extreme example would be an early calculation of $AFCR_{0}\left(t\right)$ yielding zero when the first cases have already been diagnosed up to time point *t* but no fatality has been reported up to that date.

Of note, $AFCR_{\Delta t}\left(t\right)$, even for t→∞, is not a universal classifier of a pandemic. At best, it classifies the pandemic contingent on particular local health care conditions and policies. It is particularly important in the context of COVID-19 and should therefore be emphasised that AFCR (or the case–fatality rate) is different from the so-called infection–fatality rate, since AFCR is contingent on testing coverage (cf. [16]), as mentioned in the Introduction. The same holds, of course, for Instantaneous Fatality–Case Ratio (IFCR). Assuming that the reported fatality events have previously been also reported as diagnosed cases, the fatality–case ratio can be conceived as the proportion of cases that dies.

#### 2.2.2. Diagnosis-to-Death Duration via Maximum Correlation between Deaths and Time-Delayed Cases

In order to estimate the duration from time of diagnosis to time of death, we introduce the simple approach of maximising Pearson’s correlation coefficient of the two time series, deaths(t) and cases(t−Δt) (t=Δt,⋯,T), as a function of delay time Δt or, alternatively, of ln(cumDeaths(t)) and ln(cumCases(t−Δt)). Whether the time lag between deaths(t) and cases(t), i.e., the value of Δt that optimises the delay-time correlation, yields a good approximation to the average diagnosis-to-death duration as estimated from a follow-up of individual cases until their deaths crucially depends on the presence of a salient temporal pattern in the cases(t) time series that induces a similar time shifted pattern in the deaths(t) time series. In the worst case of a homogeneous time series without epidemic ruptures, the time-delay correlation might be insensitive to detecting the diagnosis-to-death duration. In the following, we assume that the proposed method yields an acceptable approximation to diagnosis-to-death duration.

The logarithms for the cumulative data are necessary to scale data to a evaluable range. As a heuristic way to construct confidence intervals for the estimated diagnosis-to-death durations, we use the Steiger test of the difference between two independent correlations [25]. Pairwise comparisons of any correlation with the maximum correlation yields a series of *p*-values. All delays for which the correlation coefficient do not significantly differ from the maximum correlation coefficient are defined to lie within the confidence interval of the optimal estimate. Alternatively, the mutual information measure applied to the two time series could be used. However, Shannon entropy and the related Kullback–Leibler divergence, which serve as a basis for mutual information, tend to discriminate small differences relatively less in favour of discriminating larger differences of the two time series (cf. [26]).

#### 2.2.3. Generation Time via Delay-Time Autocorrelation of Cases and Deaths

Suppose that $t_{g}$ is the mean generation time of the SARS-CoV-2 virus. Cases diagnosed at time *t* should then create a second generation of cases at time point $t+t_{g}$. It might therefore be worth checking the incidence time courses for time-delayed autocorrelations, C(Δt). Trivially, the non-delayed autocorrelation should be C(0)=1. For small delays Δt, correlation C(Δt) should decline until Δt approaches the generation time $\Delta t=t_{g}$. However, a plateau or a local maximum of C(Δt) around $\Delta t=t_{g}$ is possible only for non-homogeneously distributed cases and if the variance of the generation time is relatively small. In other words, if the incidence peaks at a given point in time *t*, e.g., due to a singular event such as a mass infections at a large party, a subsequent (damped and widened) peak should be detectable at time point $t+t_{g}$.

Unfortunately, non-homogeneity in the data may also arise due to systematic delays in the diagnostic process (e.g., less tests on weekends) and delays in reporting the data: the “weekend effect”. A possible escape from the “weekend effect” could be the usage of death records instead of cases. However, by all means, the confounding “weekend effect” has to be kept in mind when evaluating delay-time autocorrelations. Furthermore, a periodogram is constructed in order to confirm the periods found by means of (auto)correlation analyses. In addition, a cross-spectrum is constructed to show the coherence between the time series of new cases and fatalities.

Based on an estimate for $t_{g}$, the ratio

(4) $$R\left(t\right)=cases\left(t+t_{g}\right)/cases\left(t\right)$$

intuitively yields a first rough estimate for the time-dependent effective reproduction ratio. Of course, the next generation of infections is in reality not created all at once after one generation time has passed, i.e., this calculation should be conceived as an orientation. Equation (4) as an approximation to R(t) can additionally be justified by assuming the counts of cases to be Poisson variates. Then, the likelihood that cases(t−Δt) counts produce $r_{\Delta t}\cdot cases\left(t-\Delta t\right)$ counts Δt days later is given by

(5) $$L=\frac{{\left(\sum_{\Delta t=1}^{t}r_{\Delta t}cases\left(t-\Delta t\right)\right)}^{cases(t)}}{cases(t)!}e^{-\sum_{\Delta t=1}^{t}r_{\Delta t}cases\left(t-\Delta t\right)}.$$

Reducing the distribution of delay-specific contributions $r_{\Delta t}$ to the reproduction R(t) at time point *t* to a single nonzero value for $\Delta t=t_{g}$ yields Equation (4) after maximising the likelihood. Of note, if variance is addressed and overdispersion supposed, the use of a negative binomial distribution rather than Poisson is indicated (cf. [27]), which is beyond our pragmatic aim. It is finally worth noting that an estimate of R(t) according to Equation (4) does not depend on the true number of infected individuals as long as the ratio of unreported to diagnosed cases is constant over time.

#### 2.2.4. Piecewise Exponential Growth and the Basic Reproduction Number

For a given time interval (t,t+Δt), the epidemic growth can be approximated by an exponential growth

(6) $$cumCases\left(t+\Delta t\right)=cumCases\left(t\right)\cdot e^{\lambda_{\Delta t}\left(t\right)\Delta t}.$$

The time-dependent rate of infection is then given by

(7) $$\lambda_{\Delta t}\left(t\right)=\frac{1}{\Delta t}\left[\ln\left(cumCases(t+\Delta t)\right)-\ln\left(cumCases(t)\right)\right].$$

Rather than $\lambda_{\Delta t}\left(t\right)$, the doubling time $t_{d}\left(t\right)$ of an epidemic phase is frequently discussed in the literature [4], which is simply $t_{d}\left(t\right)=\frac{\ln(2)}{\lambda_{\Delta t}\left(t\right)}$ for a given interval length Δt.

A well-known approximation to the basic reproduction number $R_{0}$ [4] is given by

(8) $$R_{0}=1+\frac{D\ln(2)}{t_{d}},$$

with *D* being the duration of infection, or more precisely, the duration of infectiousness. In this case, $t_{d}$ should be the doubling time of the early onset phase of the epidemic. The approximation can be derived from the analysis of an SIR model,

(9) $$\begin{array}{ccc} \frac{dS}{dt} & = & -\frac{R_{0}}{D}SI\\ \frac{dI}{dt} & = & \frac{R_{0}}{D}SI-\frac{1}{D}I\\ \frac{dR}{dt} & = & \frac{1}{D}I \end{array}$$

conditional on having a fully susceptible population (S=1) with only one index case $I_{0}$. Thus, the second equation has closed solutions $I\left(t\right)=I_{0}e^{\frac{R_{0}-1}{D}t}$ and $I\left(t_{d}\right)=2I_{0}$ after the doubling time $t_{d}$ gives the approximation in Equation (8). However, we use this formula to estimate an effective time-dependent reproduction number:

(10) $$R\left(t\right)=1+\frac{D\ln(2)}{t_{d}\left(t\right)}=1+D\lambda_{\Delta t}\left(t\right).$$

Of note, the reproduction number does not determine the duration of an epidemic. Rather, the duration is scaled via the duration of an infected individual being infectious, *D*. So far, when only using the reported incidence data of COVID-19, the magnitude of *D* is unknown. We suppose, however, that *D* is in the same order of magnitude as the generation time, if not identical (cf. [28] for a discussion of serial interval and generation time).

It must be stressed at this point that Equation (10) strictly holds only at the beginning of the epidemic. Conditional on a priori knowledge on generation time *D*, fitting the simple SIR model in Equation (9) to the early epidemic phase characterised by an absence of interventions should lead to the same estimate for $R_{0}$ due to the compatibility of the approximation in Equation (8) with the SIR model in Equation (9). Using Equation (10), R(t) has a lower bound of 1; thus, as soon as the doubling time approaches very large values, the approximation in Equation (10) for R(t) no longer holds.

The reproduction potential of the virus in a population can also be quantified by simply using the per capita growth rate $\frac{1}{cases}\cdot \frac{d(cases)}{dt}$ approximated by $\frac{1}{cases}\cdot \frac{cases(t)-cases(t-1d))}{1d}$. Along these lines, an alternative way to estimate the rate of infection with Δt=1d is given by $\frac{1}{cumCases}\cdot \frac{d(cumCases)}{dt}\approx \frac{1}{cumCases}\cdot \frac{cumCases(t)-cumCases(t-1d))}{1d}$.

## 3. Results

### 3.1. Fatality–Case Ratios Worldwide and for Eight Selected Countries

**Figure 1:**
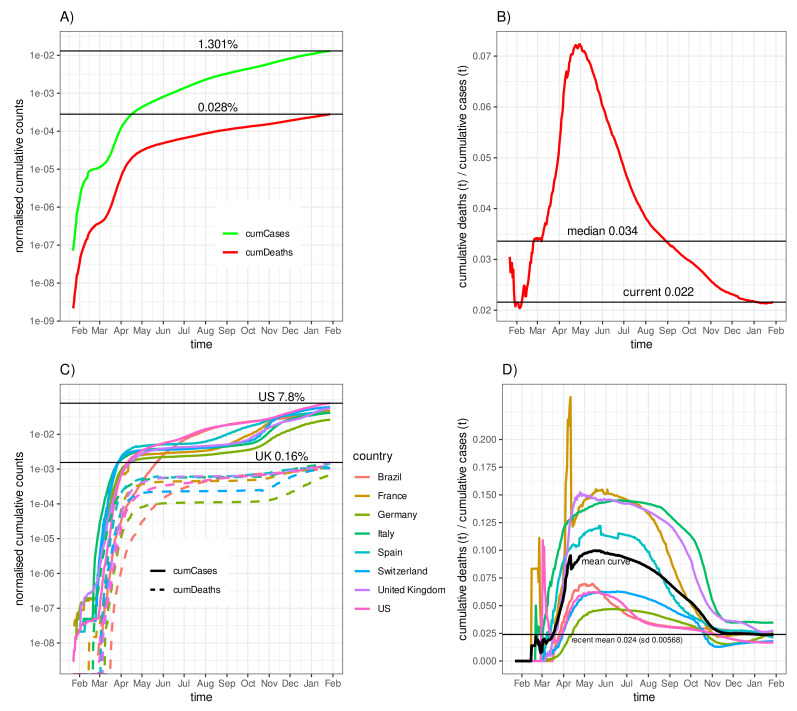
Time courses of cumulative cases, cumulative deaths, and delay−0 asymptotic fatality–case ratios for the entire world and eight selected countries: (**A**) worldwide cumulative cases and cumulative deaths normalised to the world population (per capita values), (**B**) worldwide ratio of cumulative deaths to cumulative cases (delay−0 asymptotic fatality–case ratio, (**C**) normalised (per capita) cumulative cases and cumulative deaths of eight selected countries, and (**D**) melay−0 asymptotic fatality–case ratio for the eight selected countries.

Figure 1 gives a first impression of the worldwide and a few country-specific time courses of cumulative cases and deaths. The time series have been normalised to the world population size (Figure 1A) or the corresponding country population sizes (Figure 1C). To date (28 January 2021), the worldwide proportion of diagnosed (reported) cumulative COVID-19 cases reached 1.3% of the world population size and roughly 0.028% deaths (Figure 1A), which corresponds to 2.2% of the diagnosed cases. The corresponding worldwide Delay−0 asymptotic fatality–case ratio $AFCR_{0}\left(t\right)$ (frequently denoted as the case–fatality rate in the literature) time course is shown in Figure 1B. The temporal median amounts to 0.034. However, a considerable drift can be observed. Whereas the initial variation during January might be explained as a fluctuation due to small numbers of cases and deaths, the drift from February appears to be systematic. The outbreak started in China and spread with different delays to other countries, which might at least partially play a role for the drift, particularly because countries that joined in later had different policies of testing on social contact restrictions. The enormous rise in mortality until roughly mid-May is perhaps due to overwhelmed health care systems. The subsequent decline, on the contrary, is likely due to the increasing frequency of testing for SARS-CoV-2 infections. A combination of these two effects is likely.

A glance onto Figure 1C,D confirms that different countries contributed with different relative numbers of cases and deaths to the pandemic. The current cumulative number of cases of the United States reached 7.8% and the number of deaths in the UK reached 0.16% of the population sizes as the two extremes (out of the eight countries analysed). A comparatively low incidence (of registered cases) can be observed for Germany. While Germany, the USA, and Switzerland each had a moderate case–fatality rate below the mean curve averaged over the eight countries before September 2020, France ranked highest with a median value of about 0.15; however, all case–fatality rates started to decline at the beginning of June 2020 and gradually converged to a current mean of 0.024, with a rather small standard deviation of 0.0057 taken over the eight countries (see Figure 1D). We refrain from going into depth with interpretations; however, an obvious explanation is the relatively low number of tests performed per 1000 inhabitants in France during the first half of the epidemic, as has been reported, e.g., by the Organisation for Economic Co-operation and Development (OECD) [14]. We speculate that this holds for other countries as well. It should also be mentioned that COVID-19 mortality is age-related. Thus, countries with a correspondingly age-structured demography such as Italy, with one of the oldest populations in the world, are perhaps particularly vulnerable to COVID-19 morbidity and mortality [21,29].

The sigmoid shape of the curves of the fatality–case ratios is striking. For many countries (including those not shown), the curve starts with fluctuations around a moderate value, followed by a systematic increase and eventual decline towards the end of the curve. We already discussed the impact of testing coverage. However, there is a further crucial aspect that has been neglected so far. Diagnosed individuals with a fatal course die with a certain delay after diagnosis. Therefore, shortly after the first cases are diagnosed, the fatality curve starts at zero until the first deaths occur. Therefore, we expect that the two curves, cumCases(t) and cumDeaths(t), are shifted against each other by some delay Δt such that the ratio eventually becomes constant over time for a proper choice of the delay.

### 3.2. Diagnosis-to-Death Duration for Germany

**Figure 2:**
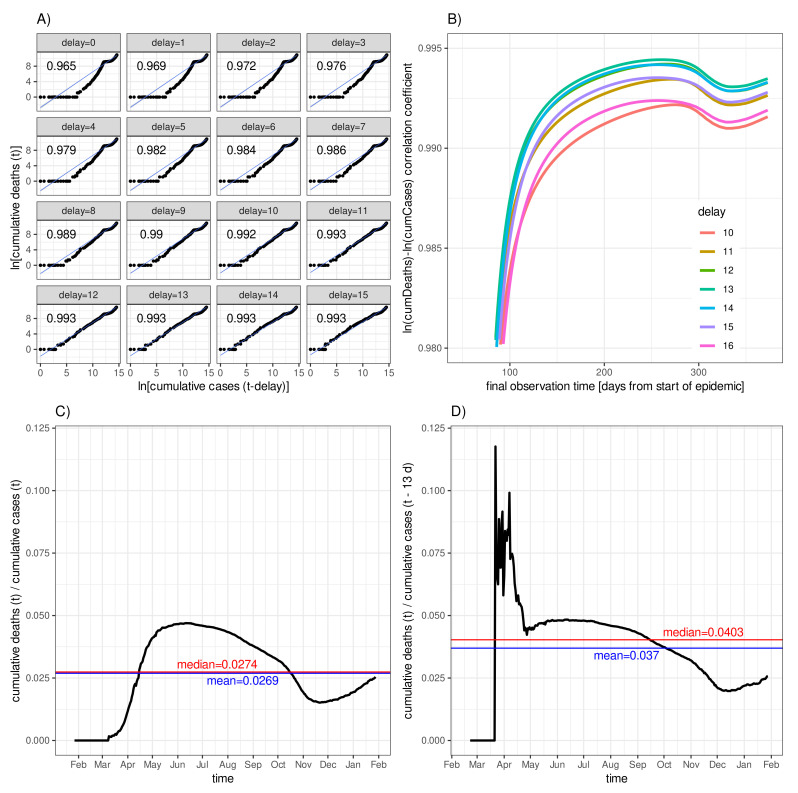
Asymptotic fatality–case ratio for the German COVID-19 data: (**A**) logarithmised cumulative deaths, $\ln\left(cumDeaths(t)\right)$, versus time delayed logarithmised cumulative cases, ln(cumCases(t−delay)), for different delays as indicated in the panel headers along with linear correlation (regression line plus Pearson’s correlation coefficient printed in the upper left corner of each panel); (**B**) correlation coefficient as a function of the lengths of the time series (i.e., final observation time) for delays ranging from 10 to 16; (**C**) delay−0 asymptotic fatality–case ratio (black) with time average (blue) and median (red); and (**D**) delay−13 asymptotic fatality–case ratio (black curve) with time average (0.037, blue line) and median (0.04, red line).

Figure 2 shows the result of a delay-dependent correlation analysis applied to the two time series cumCases(t−Δt) and cumDeaths(t) with varying delay Δt for the German COVID-19 data. The first panel, Figure 2A, shows scatter diagrams for logarithmised cumulative deaths, ln(cumDeaths(t)), versus time delayed logarithmised cumulative cases, ln(cumCases(t−delay)), for a series of 16 subsequent delays Δt=0,1,⋯,15. In addition, for each delay, the fitted line resulting from a linear regression is shown along with the values of the corresponding correlation coefficients. For delay Δt=13d, the scatter diagram transforms into an almost perfect straight line resulting in a perfect correlation coefficient that assumes 0.993. The question of whether the derived maximum correlation depends on the final observation time, *T*, i.e., on the lengths of the time series, is addressed in Figure 2B. It can be concluded from Figure 2B that, for $T-t_{0}>100d$, a delay of Δt=13d constantly turns out to yield the maximum correlation; however, the curve nearly coincides with the correlation time course for a delay of Δt=12d.

The delay−0 asymptotic fatality–case ratio according to Equation (2) is depicted in panel Figure 2C along with the time average (blue line) and median (red line). Finally, Figure 2D shows the delay−13 asymptotic fatality–case ratio along with time average (0.037, blue) and median (0.04, red) corresponding to the optimal delay of Δt=13d.

The simple time-delay correlation leads to a convincing estimate for the diagnosis-to-death duration, confirmed by comparing panels Figure 2C,D. Early after the outbreak in Germany, the delay−13 asymptotic fatality–case ratio exhibits fluctuations due to rather low counts of deaths in the beginning. After a moderate rise between May and July, the ratio dropped considerably until December 2020, which indicates a decrease in the ratio of undetected to diagnosed case numbers. The rise from December onward is perhaps attributable to extreme delays in reporting fatalities leading to spurious accumulations after the Christmas holidays [13].

One of the shortcomings of this “quick-and-dirty” approach is the lack of well-defined information on the variance of diagnosis-to-death duration. However, a heuristic indicator is given by the differences in the delay-specific correlation coefficients around the maximum, which can be tested against the null hypothesis of no difference using the so-called Steiger test [25]. Table 1 lists the estimated correlation coefficients for all delays Δt and the *p*-values resulting from testing the nullhypotheses of vanishing differences of any one correlation coefficient to the maximum coefficient, in this case, the one for delay Δt=13d. We conclude from the adjusted *p*-values that delays Δt=9d and Δt=15d can be conceived as the limits of a confidence interval for the estimated diagnosis-to-death duration of Δt=13d. Another approach would include a weighted sum over several delays of the delayed cumCases(t−Δt) in the denominator of Equation (2) or other techniques. Following Loy et al. [30], here, we trust “the power of our eyes” together with the plausibility provided by the outcome of the Steiger test.

Of note, the optimal delay for a maximum correlation between ln(cumDeaths(t)) and ln(cumCases(t−Δt)) on the worldwide scale turns out to be zero. The heuristic confidence interval based on the Steiger test stretches to Δt=4d. However, on this worldwide level, the incidence curves are much too heterogeneous to allow for reliable conclusions on the diagnosis-to-death duration. Presumably, discrimination of the impact of different delays is hampered by the huge numbers of counts, given the pronounced heterogeneity and, thus, excessive dispersion.

**Figure 3:**
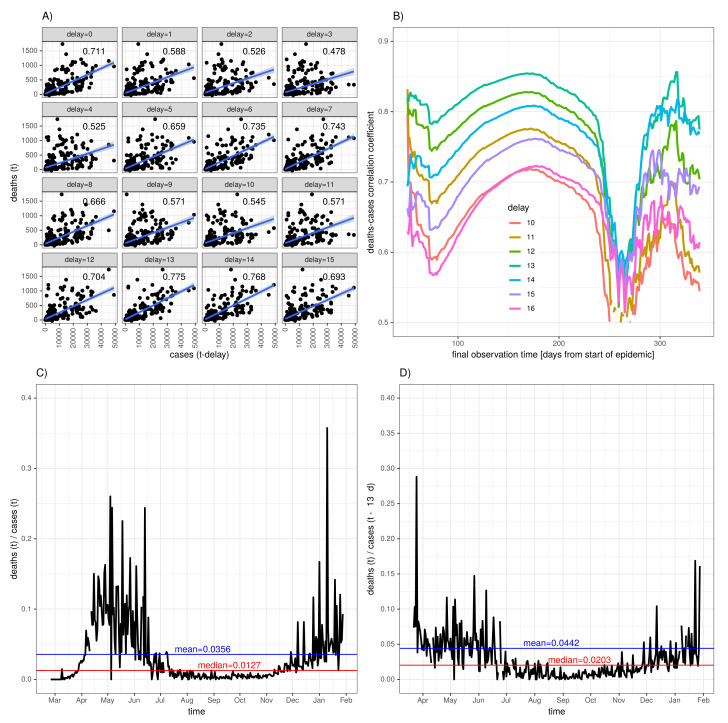
Instantaneous fatality–case ratio for the German COVID-19 data: (**A**) new deaths (deaths(t)) versus time delayed new cases (cases(t−delay)) along with linear correlation (regression line plus Pearson’s correlation coefficient printed in the upper right corner of each panel), (**B**) correlation coefficient as a function of the lengths of the time series (i.e., final observation time) for delays ranging from 10 to 16, (**C**) delay−0 instantaneous fatality–case ratio (black) with time average (blue) and median (red), and (**D**) delay−13 instantaneous fatality–case ratio (black) with time average (0.044, blue) and median (0.02, red).

In the following, the procedure of maximising correlation is applied to the German incidence time series. We expect a greater power of discriminating the delays since the application to the cumulative counts has a damping effect. Figure 3 has an analogue structure to that in Figure 2, with the incidence data replacing the cumulative incidence.

Panel Figure 3A shows deaths(t) versus cases(t−delay) for a series of 16 subsequent delays Δt=0,1,⋯,15 (in days). For each delay, the fitted line resulting from a linear regression is shown along with the values of the corresponding correlation coefficients. For delay Δt=13d, the two time series correlate best with the correlation coefficient assuming the value 0.775. Figure 3B shows that time series comprising more that 100 days lead to robust results with the exception of passing through the October data, i.e., the derived optimal delay is not contingent on the final observation time. The intermediate loss of correlation can be attributed to the low incidence interval during summer time.

The delay−0 instantaneous fatality–case ratio according to Equation (3) is depicted in panel Figure 3C along with the time average (blue line) and median (red line). Finally, Figure 3D shows the corresponding delay−13 instantaneous fatality–case ratio along with the time average (0.044, blue) and median (0.02, red). The application of this maximum correlation variant gives us the same delay as previously estimated for the cumulative counts. Similar to that before for the cumulative incidence data, we compare the correlation coefficients by applying Steiger’s test. The result is shown in Table 2. From the *p*-values, we construct a confidence interval around the estimated delay Δt=13d ranging from Δt=12d to Δt=14d.

As expected, the instantaneous fatality–case ratio shows a more pronounced fluctuation when compared to the corresponding asymptotic fatality–case ratio. However, it is the measure of choice when time dependency of the fatality risk is the case in point. The time series of deaths may exhibit independent fluctuation; however, a hypothetically temporarily constant fatality–case ratio implies that the temporal variation of the time course of deaths follows the fluctuation of the case incidence curve, albeit with some delay. This gives us the rationale behind the assumption that maximum correlation applied to incidence data allows for a more sensitive discrimination of delays. As observed for the delay−13 asymptotic fatality–case ratio (Figure 2D), the delay−13 instantaneous fatality–case ratio (Figure 3D) remains approximately constant from May until the beginning of July, followed by a marked drop towards a lower but again approximately constant level until December, followed by a gradual increase during the winter season. This striking result leads us to conclude that the ratio of undetected to diagnosed cases dropped early July. In addition, the increasing coverage of tests applied to children most likely changed the age-structure of the (diagnosed) population. The rise during the winter season is presumably associated with delays in reporting the cases [13]. In the following, this scheme is applied to data from a set of selected countries.

### 3.3. Diagnosis-to-Death Duration for the Eight Selected Countries

**Figure 4:**
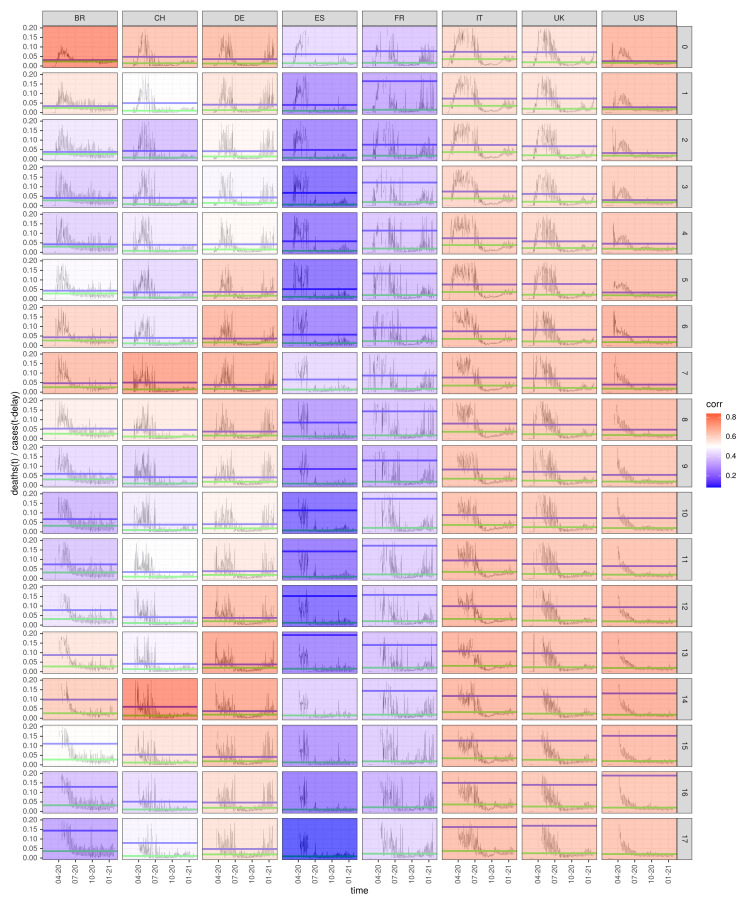
Diagnosis-to-death duration for eight selected countries analysed using delay-time correlation: the plot shows the magnitudes of delay-specific correlations between deaths(t) and cases(t−Δt) for the eight selected countries (column labels) in the form of a heatmap. The delays Δt (row labels) run from Δt=0d through Δt=17d. Strong correlations are shown in dark red, and declining correlation coefficients gradually fade to blue. Also shown for each country and each delay are the time courses of Delay−Δt instantaneous fatality case ratios along with time average (blue line) and median (green).

In this section, a comprehensive summary plot of the diagnosis-to-death durations for the eight selected countries is presented and discussed (Figure 4). The same algorithm as discussed for Germany in the previous section is applied to seven further countries (France, Italy, Spain, Switzerland, the UK, the USA, and Brazil). Concretely, for each of the eight countries’ incidence data, a series of coefficients for the correlation between deaths(t) and cases(t−Δt) is calculated with delays Δt ranging from 0 to 17 days. The results are depicted in Figure 4 in the form of a heat map. Each column of the panel array represents a country, as denoted in the top panel labels. The series of 18 delays for each country is displayed in the vertical direction as indicated by the right-hand vertical labels. The magnitudes of the correlation coefficients are colour coded. A glance onto the second column confirms the findings from the previous section: the colour saturation peaks for the delay Δt=13d for the German incidence data, which lead us to conclude that late individuals survived in the average 13 days after their COVID-19 diagnosis.

For some countries, such as for the USA, the UK, and Italy, the correlation coefficients remain at a moderate level for all delays. For these countries, a less marked maximum at delay Δt=13d can be observed. The flat distribution of the magnitudes of the correlation coefficients for the States likely reflects the heterogeneity of sampling incidence data (e.g., spatially as well as temporarily nonconstant testing coverage). The same holds for the UK and Italy. The distribution of diagnosis-to-death durations of all countries weakly peak at very small delays and are somewhat more pronounced at 7 days, which is most likely due to the spurious “weekend effect”.

At this point, we confer [24] with a final observation time in early September. In particular, an analysis restricted to the “first wave” of the pandemic points to a test procedure where infected persons are diagnosed rather late and only with severe symptoms. This particularly holds for Spain and Italy. Therefore, the distributions of diagnosis-to-death durations for these countries peak at very small delays of around 2–4 days during the “first wave” (results not shown). Again, we refrain from going into depth with interpretations. Of note, however, are the short survival times after diagnosis for Italy and Spain, an insight that is confirmed with reports on overwhelmed public health authorities and the generation spanning human-to-human social contact behaviour [14,21,29]. Also of note, amongst the eight countries selected, Germany and Switzerland have the longest and at the same time most reliable diagnosis-to-death durations. Of note, this result strongly depends on the population size and may vary when looking at more homogeneous subpopulations such as states and counties.

**Figure 5:**
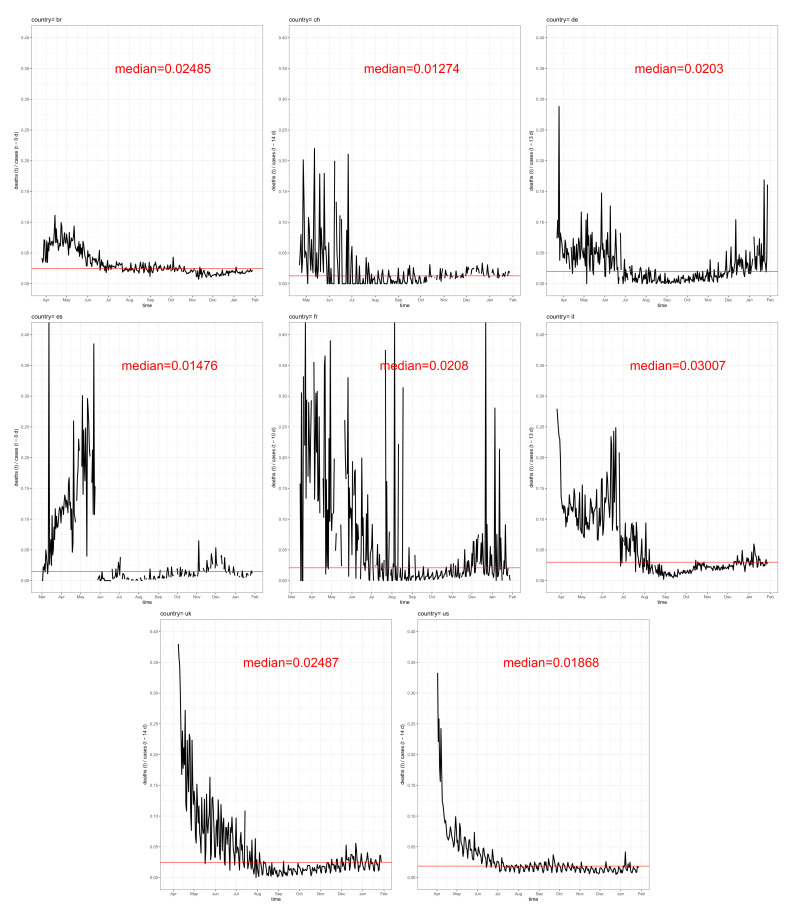
Instantaneous fatality–case ratios stratified for the analysed 8 exemplary epidemics: the corresponding country code is assigned to the top of each panel.

The most striking result of our analysis is depicted in Figure 5. Using the optimal delays Δt for each of the eight selected countries yield $IFCR_{\Delta t}\left(t\right)$ time series differing considerably less than expected from mass media reports. However, the role of the proportion of undetected infections as well as the country-specific age-distribution remains unclear due to a lack of available data. Of note, the early phases are characterised by strong fluctuations and high levels of fatality–case ratios thus strongly bias the median. Apparently, the testing frequencies maximally differed between the eight countries during the early phase of the pandemic. Unfortunately, authorised and reliable data on frequencies of testing are rare for most countries. From bulletins of official authorities and WHO or OECD reports (available from the websites of the organisations, e.g., [14,31,32]), it is at least possible to vaguely reconstruct that Switzerland has a comparably high COVID-19 test frequency compared, e.g., with Brazil and Italy. Therefore, the fact that Switzerland ranks lowest with respect to the median of the instantaneous fatality–case ratio (0.013) and that Italy and Brazil have higher ratios (0.03 and 0.025) might be due to the differences in test frequencies. The fact that Italy is one of the oldest countries in terms of age-distribution, leading to a large proportion of vulnerable individuals, should also be considered.

### 3.4. Negative Correlation of the Fatality-to-Case Ratio with the Number of Cases

Interestingly, the instantaneous fatality–case ratios, $IFCR_{13d}\left(t\right)=\frac{deaths(t)}{cases(t-13d)}$, both for Germany and Italy exhibit negative correlation with the number of 13-day delayed newly confirmed cases, cases(t−13d). However, this correlation presumably reflects a time effect rather than a genuine incidence effect due to several reasons. The increasing frequency of testing as one of the suspected confounders has already been mentioned. Furthermore, most vulnerable individuals presumably have been infected and passed away in the early phase of the pandemic, particularly in Italy. In addition, an adaptation and an improvement in the health care system and advances in medicine appear to be plausible. The temporal effect is obvious from throwing a glance on Figure 3D, anyway. However, disentangling incidence and temporal effects appear to be difficult in view of the sparse available information.

A linear regression, taking the interaction between incidence and time into account, yields results as shown in Table 3. The interaction neither for Germany nor for Italy compensate for the negative slopes of the two main effects. However, the effects are all negligible for Germany but not so for Italy. At a first glance, this result apparently contradicts the conjecture of an increasing number of deaths due to an overwhelmed health system. However, it has to be interpreted with caution as long as the correlation with the number of severe cases and hospitalisation is missing. Also important, a linear regression is certainly not adequate to capture the obviously nonlinear temporal effect. We refrain from expanding upon this aspect here; however, it should be considered in greater detail in future studies.

### 3.5. Estimating Generation Time

**Figure 6:**
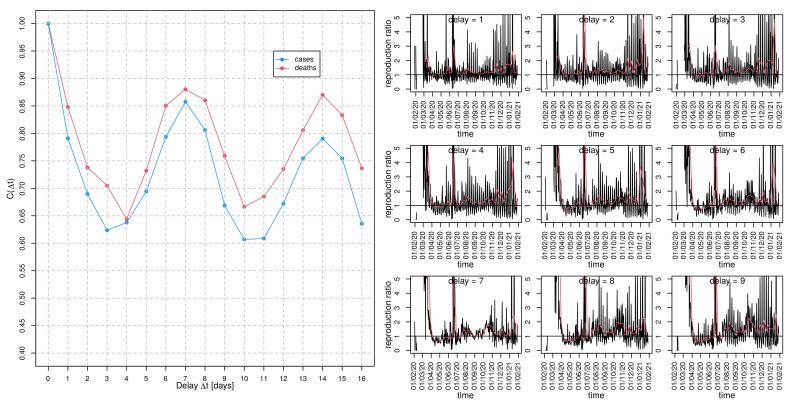
Delay-time autocorrelation for German incidence data: (**left** panel) autocorrelation, C(Δt), of cases (blue curve) and deaths (red) as a function of delay Δt and (**right** panel) ratio $\frac{cases(t)}{cases(t-\Delta t)}$ (primitive approach to estimate the reproduction ratio) for nine different delays Δt, as indicated in the panel headers. The red curves result from a moving average with a window width of seven days.

The result of the delay-time autocorrelation C(Δt) of both cases(t) as well as deaths time series, i.e., the correlations between cases(t) and cases(t−Δt) as well as the corresponding fatalities, respectively, for the German data are depicted in the left panel of Figure 6. The autocorrelation of the fatality time series shows a decent plateau roughly between delay Δt=3d and Δt=4d and both curves peak at Δt=7d. A further plateau is visible for both curves at Δt=14d. While a generation time between 3 and 6 days appears to be plausible [28], the observed peak at Δt=7d could also be the impact of the “weekend effect” (e.g., aggregated counts from the weekend on Monday which were not retro-corrected).

Whereas the COVID-19 testing frequency might be substantially lower on weekends, leading to a biased peak of Monday or Tuesday incidence, the occurrence of fatalities should not depend on the weekday. However, there are also substantial delays in reporting [13]. Unfortunately, although there exist claims of assigning occurrences to the correct date, an assurance is not possible. Having said that, the pronounced local maxima of the delay-time autocorrelation C(Δt) of the deaths(t) time series at Δt=7d and Δt=14d are striking. The estimation of the preliminary instantaneous “reproduction ratio” for Germany according to Equation (4) with the delay $t_{g}$ varying between one and nine is depicted in the right panel of Figure 6. A visual inspection of the produced curves clearly shows that delay $t_{g}=7d$ leads to the best reduction of noise, pointing to the superiority of $t_{g}=7d$. Apparently, during April and May, the contact restrictions had been successful since R(t) remains considerably below 1 during this episode. Starting in June, R(t) exceeded again the threshold of 1, which caused an increased instantaneous incidence and gave rise to the so-called “second wave” during the winter season.

**Figure 7:**
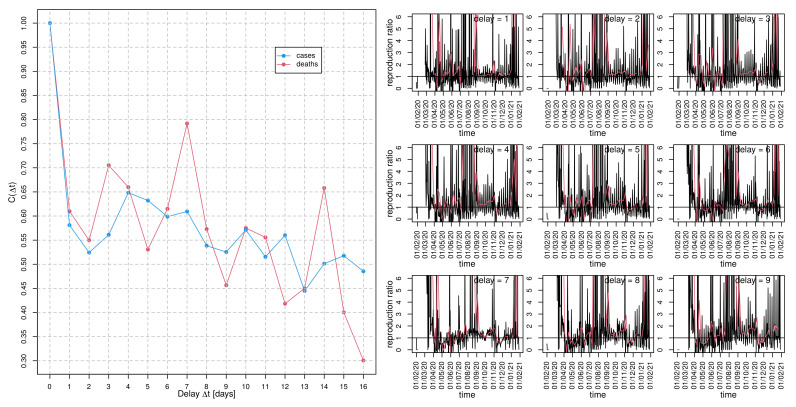
Delay-time autocorrelation for French incidence data: (**left** panel) autocorrelation, C(Δt), of cases (blue curve) and deaths (red) as a function of delay Δt and (**right** panel) ratio $\frac{cases(t)}{cases(t-\Delta t)}$ (primitive approach to estimate the reproduction ratio) for nine different delays Δt, as indicated in the panel headers. The red curves result from a moving average with a window width of seven days.

For the French incidence data, local maxima of the correlation function at almost identical time delays can be observed (see left panel of Figure 7)and, in fact, was even more pronounced than for the German data. Again, the noise is maximally reduced for delay $t_{g}=7d$ for the preliminary instantaneous “reproduction ratio” for France (cf. right panel of Figure 7). In comparison to the German time course of R(t), the French instantaneous reproduction ratio is noisier and exhibits rather strong occasional bursts even during the moderate epidemic activity from May on.

It goes without saying that we have to be cautious with conclusions. However, if the observed periodicity results from the weekend effect, it entails an urgent need for quality management of data acquisition since a correct assessment of the COVID-19 epidemic data is pressing. To which proportion the generation time and the weekend effect contribute to the observed “resonance” in the delay-time correlation remains an open issue.

Despite the aforementioned uncertainties, the suggested methodological approach remains noteworthy. Moreover, independently from its cause, the observed periodicity is important for the assessment presented in the following section.

### 3.6. Time-Dependent Infection Rate and the Effective Reproduction Number

**Figure 8:**
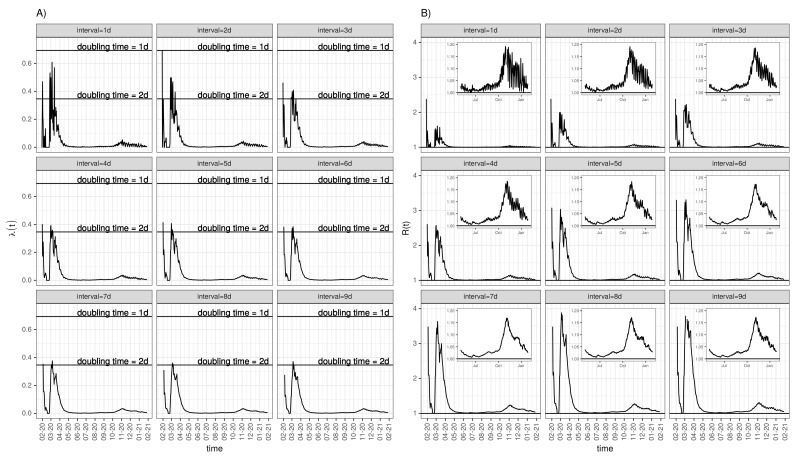
Time-dependent infection rate and approximate effective reproduction number for Germany: (**A**) time course of the infection rate $\lambda_{\Delta t}\left(t\right)$ according to Equation (7) for nine different intervals (delays) Δt, as indicated in the panel headers. Also shown are lines that correspond to doubling times of either 1d or 2d, respectively. (**B**) Approximate reproduction numbers calculated according to Equation (8). The inlets show details where *R* is close to 1, i.e., from May onwards. Of note, computed this way, *R* has a lower limit of 1.

The instantaneous infection rate (Equation (7)) of the German epidemic, assumed to be piecewise constant over short time intervals of length Δt days, is depicted in Figure 8A for a series of nine intervals from one through nine days. Once more, the interval of seven days appears to be an optimal choice with respect to noise reduction due to the corresponding periodicity of the incidence time series. If the initial phase before March is skipped due to the uncertain estimation resulting from relatively few counts, the peak in early March can be conceived as a good approximation to the initial infection rate of the epidemic. This peak value corresponds to a doubling time of two days. Using Equation (8) to calculate an approximate $R_{0}$ yields $R_{0}=1+\frac{D\ln(2)}{t_{d}}=1+\frac{\ln(2)\cdot 7d}{2d}=3.4$, where we set D=7d due to our findings above. However, if we use a four-day generation time instead, motivated by the moderate plateau emerging at about Δt=4d in the delay-time autocorrelation depicted in the left panel of Figure 6, then $R_{0}$ assumes 2.4.

The entire time series R(t) calculated according to Equation (8) for all nine chosen intervals Δt=1,2,⋯,9 are shown in Figure 8B. It is worth to emphasise again that R(t) computed this way is reliable only for values well above 1. Strictly speaking, Equation (8) is an approximation to $R_{0}$, i.e., the basic reproduction number defined for the early epidemic phase.

### 3.7. Per Capita Growth Rate as an Alternative for the Reproduction Number

**Figure 9:**
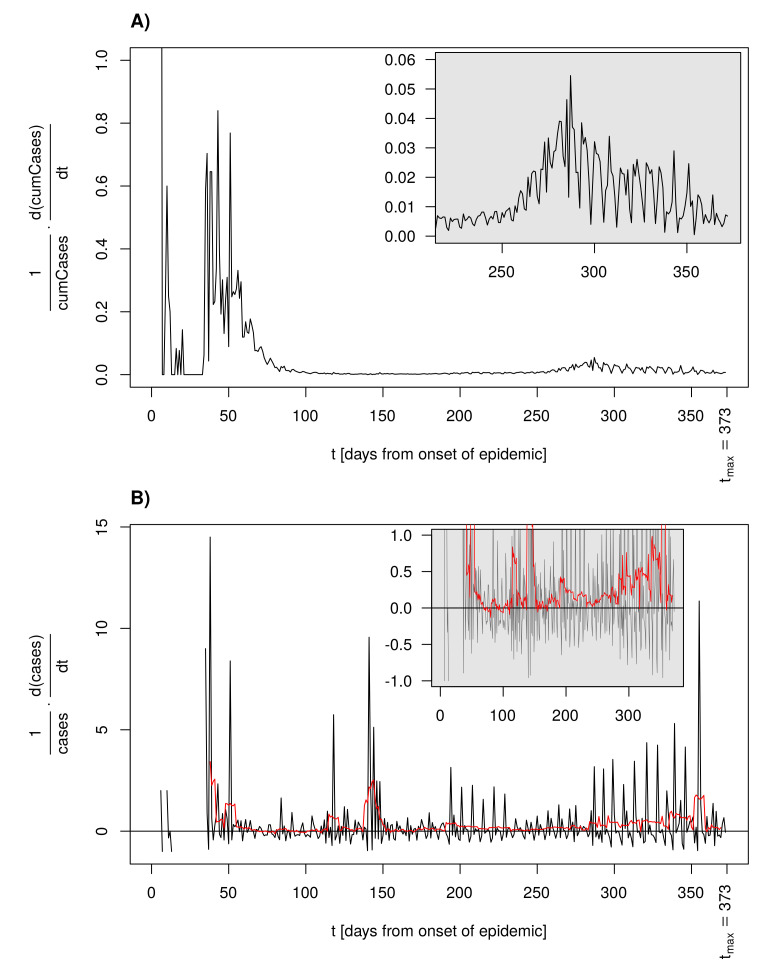
Per capita growth rates by time for the German COVID-19 data: (**A**) growth rate for cumulative cases, where the inlet shows the tail of the time course for t>220d with adjusted y-axis for better visibility, and (**B**) growth rate for the daily new cases, where the inlet shows the same time course with a narrow y-axis range around zero. Red curve: moving average with a 7 day window size.

The per capita growth rates $\lambda =\frac{1}{cumCases}\cdot \frac{d(cumCases)}{dt}$ and $\alpha =\frac{1}{cases}\cdot \frac{d(cases)}{dt}$ allow for an alternative assessment of the reproduction potential of an epidemic. Per capita growth rate λ relates to the analysis presented above (cf. Figure 8, delay = 1d). Initially, i.e., for S=1, the per capita growth rate α relates to the basic reproduction number using $R_{0}=\alpha D+1$, with *D* being the generation time. The advantage is that knowledge of the precise generation time is not necessarily needed to draw inferences from α. Figure 9 shows the time course of an approximation $\hat{\alpha }\left(t\right)=\frac{1}{cases(t)}\cdot \frac{cases(t)-cases(t)-1d}{1d}$ to α. Positive α leads to epidemic growth, whereas α<0 gives rise to a decline in the size of the infected subpopulation. The moving average shown as a red curve in Figure 9B with window width equal to a week reveals that α rarely assumed a negative value in the course of the German COVID-19 epidemic. However, an estimation of instantaneous α at time points *t* with a very low number of cases(t) may become unreliable.

### 3.8. Spectral Analysis to Confirm Periods

**Figure 10:**
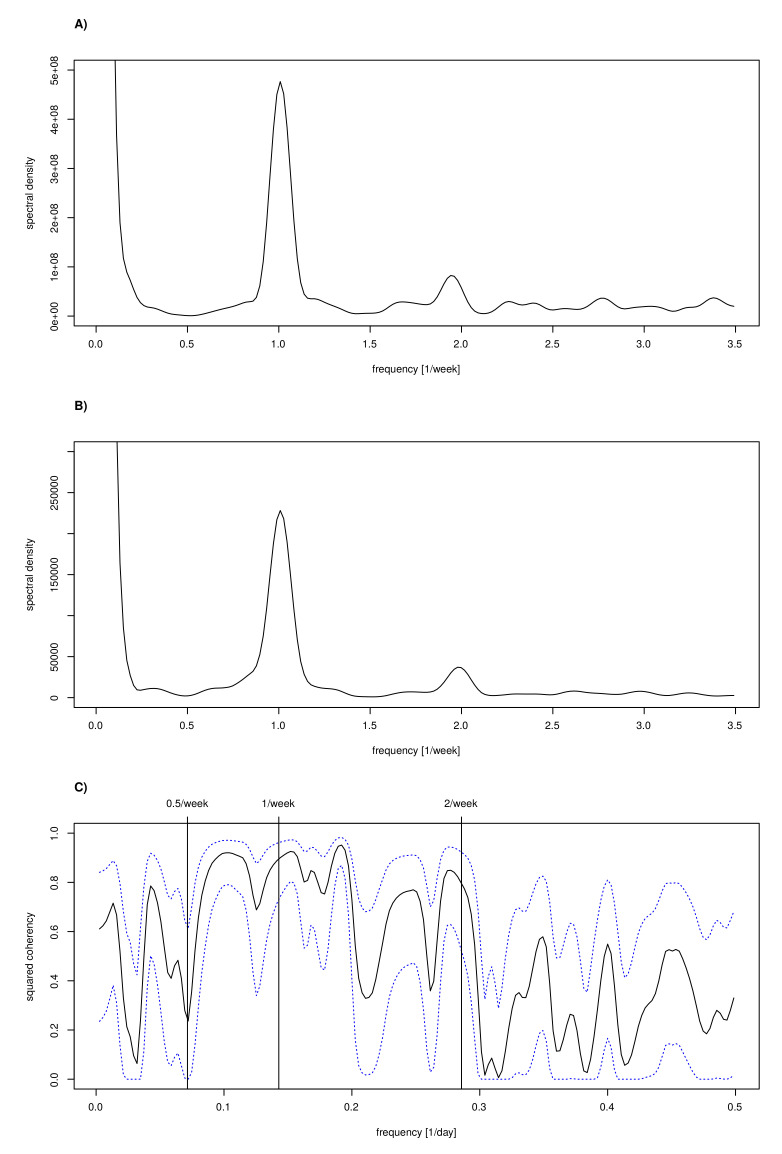
Spectral analysis for the German COVID-19 data: (**A**) spectral density of confirmed cases time series; (**B**) spectral density of confirmed deaths time series; and (**C**) cases–deaths coherency, showing the correlation at different frequencies (cross-sprectrum).

Spectral analysis is an alternative method for backing the results of delay-time autocorrelations, applicable to sufficiently long time series, which embrace several periods of interest such as the generation time. Both of the two spectral densities for the cases $S_{c,c}\left(f\right)$ and the fatalities $S_{d,d}\left(f\right)$, as depicted in Figure 10A,B, reveal two major periods. As already discussed in the context of autocorrelations, the observed one-week period is most likely attributable to the delays in reporting cases during weekends (“weekend effect”). The second observed period of approximately four days is arguably attributable to the generation time. This finding is fully consistent with findings reported in [28].

With $S_{d,c}\left(f\right)$ being the cross-spectrum of cases, c, and fatalities, d, the coherence between the two time series as defined by $\frac{|\langle S_{d,c}\left(f\right)\rangle {|}^{2}}{\langle S_{c,c}\left(f\right)\rangle \cdot \langle S_{d,d}\left(f\right)\rangle }$ and depicted in Figure 10C is striking. A strong coherence for periods between four days and roughly two weeks can be observed. Speculatively, the low frequency peak in coherence might be attributable to the “two waves” of the epidemic.

## 4. Discussion and Conclusions

Although it is a methodological challenge to derive parameter values relevant to understanding the pandemic from pure incidence data without prior knowledge from independent studies on the magnitude of some of the parameters, we attempted to evaluate incidence data without such a priori knowledge. The rationale behind this enterprise was to refrain from questionable assumptions, in particular, in the absence of proper studies that supply evidence to such assumptions.

In this article, we largely took a descriptive stance and reported the figures resulting from the estimations as they are without in-depths interpretations. Occasionally, we suggested possible obvious interpretations. For example, conditional on not surviving the infection (i.e., not in the sense of a censored survival analysis), the average time-to-death after diagnosis (or diagnosis-to-death duration) in Germany is about 13 days. During the so-called “first wave,” the time-to-death in Italy appeared to be rather short (≃2 days), albeit with very low confidence. This could be due to the different health system conditions for the two countries being worse for Italy. An even more pessimistic interpretation is to assume the diagnosis to be contingent upon death for some cases. A better understanding likely results from including demographic conditions [21,29,33], which was however not considered here. Including the “second wave” in the analysis, confidence was further lost for Italy, the USA, and the UK, i.e., the time-delay correlation function between cases and fatalities remained rather flat. We conclude that the populations particularly of these three countries as well as their local public health conditions are very heterogeneous such that the coherence between time series of cases and fatalities is lost on the country level.

Amongst the eight exemplarily analysed countries, France showed the highest fatality–case ratio during the first half of the epidemic, temporarily being close to 0.2, followed by the UK and Italy, having fatality–case ratios that peak at about 0.15, and the corresponding value in Spain was temporarily assumed at about 0.12. Until August 2020, the United States, Germany, and Switzerland had values close to the worldwide average of about 0.032. Besides the country-specific conditions of health care, another obvious interpretation is the difference in testing coverage, which might explain the huge fatality–case ratio in France, who started with mass tests not before May 2020 [14].

In the long run, the overall picture changed substantially. The case–fatality ratios of the eight countries converged to a rather narrow range around the mean value of 0.024. The most plausible explanation may be the convergence of coverage and frequency of testing. Remarkably, the more appropriate instantaneous fatality–case ratios of the eight countries remain within a narrow range of temporal medians between 0.013 and 0.03 during the second half of the pandemic. With due caution according to uncertainties in official reports, we conclude that the width of this range is more likely explained by differences in frequencies of testing than by “true” differences in COVID-19-related mortality. However, the demand for more evidence derived from proper studies is obvious.

In this context, country-specific differences in COVID-19 mortality are not exclusively explainable by differing test frequencies as suggested by the so-called excess mortality. Excess deaths, i.e., fatalities that add to the long-term temporal average related to the pandemic, are less frequent in Germany compared to the other seven countries (cf. [34,35]) included in the present study. Factors are differences in the countries’ sociocultural and demographic backgrounds as well as the conditions of the health care systems, but biological causes such as different virus strains and immunological conditions (e.g., due to differing general vaccination status) also cannot be excluded. An in-depth analysis is beyond the scope of the present study.

The calculation of fatality–case ratios using time lags between cumulative cases and deaths turned out to have substantial superiority when being compared to the commonly reported case–fatality rates. This is particularly true for estimates derived well before the epidemic comes to a halt. The correlation-based determination of an optimal time lag gives a good estimate for the average diagnosis-to-death duration and, at the same time, allows for a reliable calculation of the instantaneous fatality–case ratio. In the case of a constant testing coverage, i.e., constant ratio of undetected-to-diagnosed cases, the delayed fatality–case ratio should also be constant over time, which is what we observed for the second halves of epidemic data with the exception of Germany and France. For the latter two countries, the rise in fatality–case ratio during the winter session can be explained by a lower test frequency after the Christmas holidays and substantial delays in reporting the cases.

For most of the countries including Germany, deaths appear 10–14 days delayed with respect to the dates of diagnoses. The shortest duration of less than 3 days was estimated for Brazil and Spain. The median asymptotic delay−13 fatality–case ratio for Germany calculated as the ratio of the 13-day delayed cumulative fatality time series to the time series of cumulative cases assumes 0.04. Using the instantaneous instead of the cumulative incidence data confirms these findings and, arguably, improves the quality of the estimations because damping effects that result from cumulative summation are avoided. Remarkably, the median delay−13 instantaneous fatality–case ratio for Germany in this case assumes 0.02. This rather low value, compared to the arithmetic time average of 0.044, points to a nonnormal distribution since the mean instantaneous fatality–case ratio should always dominate the corresponding asymptotic value due to $\frac{1}{T-\Delta t}\sum_{t-\Delta t=0}^{T}\frac{deaths(t)}{cases(t-\Delta t)}\leq \frac{cumDeaths(T)}{cumCases(T-\Delta t)}$ (cf. [36]). In fact, the distribution of fatalities is extremely right-skewed due to an increased frequency of zero events during the summer season.

In a recent paper [27], our delay correlation method was applied in a geospatial way to the case and fatality counts per US county. In this case, county-specific fatality counts at a given point in time were correlated to the county-specific time-delayed number of cases. Remarkably, the authors derived the same optimal delay of 13 days that maximises correlation. This suggests a self-similar spatiotemporal process that becomes manifest in a self-similar spatial structure [37,38,39]. In many biological and societal systems, it is known that growth processes are driven by metabolic support through the surface or are characterised by a gradient of supply towards the centre of the growth unit giving rise to allometric invariance/scaling [40,41]. Along these lines, initial spots of infections heterogeneously distributed over a huge population may lead to an epidemic spread that resembles fractal growth that generates self-similar patterns unveiling in power-law distributions of the number of confirmed cases [42] and may follow a fractional SIR dynamics [19]. An in-depth analysis is planned.

Attempts to estimate the generation time based on autocorrelation of both cases as well as deaths time series is hampered by the superposition of a suspected weekly periodicity (“weekend effect”). We observed a weak increase in autocorrelation of the German data for a delay of 3–4 days and a stronger increase for a delay of 6–7 days. For the French data, the 3-day-resonance is considerably more pronounced. However, a 3.5 day periodicity for the German incidence time series could be confirmed by applying a spectral analysis.

The generation time is needed to calculate either the basic or the effective (instantaneous) reproduction number. R. Mikut et al. [43] recommend in their approach of estimating the reproduction number to use filter techniques to reduce the weekly periodicity. Such a strategy, however, risks filtering out epidemic-relevant delay effects. In their subsequent calculation of R(t), the authors adopted the generation time from other studies’ results.

Here, we presented two alternatives for estimation of the reproduction number R(t). The first version is similar to the one used by Mikut et al. [43], though without filter. Simply put, R(t) is calculated as the ratio of counts of new cases at time *t* to the number of cases at time-lagged time *t* minus the generation time $t_{g}$. We presented graphs for a series of time lags $t_{g}$ including the “hot” candidates $t_{g}≃4d$ and $t_{g}≃7d$. We observed the following: in the proximity of R(t)=1, the moving average of all time courses are almost identical, i.e., independent from the chosen $t_{g}$. The chosen time lag has a considerable effect on noise, though with minimum noise for $t_{g}=7d$. For values of R(t)≫1, the magnitude of noise prevents deriving a reliable estimate, independently from the chosen generation time. We cautiously conclude that R(t) calculated this way gives sufficiently accurate information on the magnitude of R(t) close to 1, which is of relevance for public health decision making. Therefore, opting to use $t_{g}=7d$ acts as a noise filter whether this time lag is a real epidemic, a sampling effect, or a mixture of both. An estimate of $R_{0}$ based on this approach is questionable, which obviously holds independently from the chosen time lag. In this context, it is worth noting that published estimates of $R_{0}$ vary in a range between 2.2 and values well above 5 (see, e.g., [8,43,44] and citations therein). Khailaie et al. [8] presented a similar analysis of the time course of R(t), with values at the beginning of the epidemic in Germany that even exceeded ten in some federal states. In essence, Khailaie et al. came to the same conclusion that a reliable estimate of the basic reproduction number $R_{0}$ is hampered by early interventions into the epidemic; thus, only calculations of R(t) for time points t≫0 have, for the time being, enough confidence to draw policies upon. Unfortunately, a unique definition of an effective reproduction number R(t), consequently, a “gold standard” algorithm to estimate R(t), does not exist. To put it straight, the current reproduction number is only one of a plethora of aspects that add to a control policy to combat COVID-19 such that the strategy taken is not sensitive to the chosen algorithm. This particularly holds if R(t) informs on policy in a binary way, i.e., R(t)≷0.

The second proposed version to calculate R(t) has been based on an intermediate step of firstly computing the instantaneous rate of infection. In simplified terms, we modelled the growth of cumulative cases by means of piecewise exponentials with exponents—the infection rates—held constant within subsequent time windows of equal length. We learnt from varying the length of time windows that, once more, an interval of seven days leads to a relatively smooth curve. The time course of the thus derived instantaneous infection rate translates into a time-dependent reproduction rate based on a well-known formula [4] that is compatible with a simple SIR model. In contrast to the first version above, this second way of computing R(t) yields reliable estimates for R(t) well above 1, i.e., particularly during the onset of the epidemic before interventions unfold their impact. Along these lines, the estimate of $R\left(t=0\right)=R_{0}$ coincides with an estimate derived from fitting an SIR model to the incidence data at the outset of the epidemic. Thus, this second version perfectly complements the first version above. However, the still existing uncertainty in choosing proper intervals for the stepwise exponentials entails uncertainty with respect to the correct value of $R_{0}$. Having said that, our analysis yields an approximate range $2.4<R_{0}<3.4$, which arguably adds evidence to published similar results.

An alternative way to assess the current reproduction potential of an epidemic is given by simply estimating the instantaneous per capita growth rate of the incidence. This method gives a quick but nonetheless feasible basis for decision making without knowledge of the generation time. The guiding principle for interventions is to push the per capita growth rate into the negative range. Here, we used the per capita growth rate to back our findings.

Of note, the results vary between different published datasets. Specifically, we refrained from using the fatalities recorded by the RKI due to the confusing registration dates. Apparently, the registration date corresponds to the date of the diagnosis rather than death, which can be learnt only from discussions in the comment section [12] but not from the instruction legend. Moreover, the first reference date 15 January 2020, as it appears in the RKI dataset, is inconsistent with findings published in [6], which dates the earliest possible effective contact back to January 20. However, rigorously assessing the quality of data curation is far from straightforward and beyond the intention behind the presented analysis.

In conclusion, we emphasise that, here, we backed our arguments in part using heuristics and gained insight by trusting in the “power of our eyes” [30], an approach occasionally called “quick and dirty.” Our aim was to prepare for more complex mathematical modelling with an initially autonomous estimation of temporarily changing parameters, thus backing our arguments on comprehensible algorithms straightforwardly applied to pure incidence data. We very much hope to enrich the existing discourse on this hazardous COVID-19 pandemic.

## Figures and Tables

**Table 1:** Comparison of correlation coefficients for the cumulative incidence data: column 2 contains the estimated correlation coefficients of the two time series ln(cumCases(t−Δt)) and ln(cumDeaths(t)) with the corresponding delays Δt in days listed in the first column. The *p*-values in the third column refer to a test for difference of any given correlation coefficient with the maximum correlation coefficient, in this case, the one estimated for delay Δt=13d. The last column contains the corresponding Benjamini–Hochberg adjusted *p*-values. Some *p*-values assume 0.000 after rounding; thus, p<0.0005 in such cases.

Delay	Corr	*p*	*p*_adj
0	0.965	0.000	0.000
1	0.969	0.000	0.000
2	0.972	0.000	0.000
3	0.976	0.000	0.000
4	0.979	0.000	0.000
5	0.982	0.000	0.000
6	0.984	0.000	0.000
7	0.986	0.000	0.000
8	0.989	0.000	0.000
9	0.990	0.007	0.112
10	0.992	0.085	1.000
11	0.993	0.416	1.000
12	0.993	0.839	1.000
13	0.993	1.000	1.000
14	0.993	0.855	1.000
15	0.993	0.506	1.000

**Table 2:** Comparison of correlation coefficients for the incidence data: column 2 contains the estimated correlation coefficients of the two time series cases(t−Δt) and deaths(t) with the corresponding delays Δt in days listed in the first column. The *p*-values in the third column refer to a test for difference of any given correlation coefficient with the maximum correlation coefficient, in this case, the one estimated for delay Δt=13d. The last column contains the corresponding Benjamini–Hochberg adjusted *p*-values. Some *p*-values assume 0.000 after rounding; thus, p<0.0005 in such cases.

Delay	Corr	*p*	*p*_adj
0	0.711	0.065	0.087
1	0.588	0.000	0.000
2	0.526	0.000	0.000
3	0.478	0.000	0.000
4	0.525	0.000	0.000
5	0.659	0.002	0.004
6	0.735	0.234	0.288
7	0.743	0.335	0.383
8	0.666	0.003	0.005
9	0.571	0.000	0.000
10	0.545	0.000	0.000
11	0.571	0.000	0.000
12	0.704	0.045	0.065
13	0.775	1.000	1.000
14	0.768	0.826	0.881
15	0.693	0.023	0.037

**Table 3:** Linear regression Instantaneous Fatality–Case Ratio (IFCR) by cases × time. The standard errors of the estimates are in parentheses.

	IT	DE
(Intercept)	0.158 ${}^{***}$	0.054 ${}^{***}$
	(0.011)	(0.014)
1000 cases	$-0.009{}^{\;***}$	−0.005
	(0.003)	(0.004)
time (months)	$-0.020{}^{\;***}$	−0.002
	(0.002)	(0.002)
cases:time	$0.001{}^{\;***}$	0.001
	(0.000)	(0.000)
R${}^{2}$	0.329	0.011
Adj. R${}^{2}$	0.322	0.001
Num. obs.	303	307

*** *p* < 0.001.

## Data Availability

Only open access data from Johns Hopkins University have been used: https://github.com/CSSEGISandData/COVID-19/tree/master/csse_covid_19_data/csse_covid_19_time_series, (accessed on 18 March 2021). The R source code is provided at https://github.com/Diebner/Nonparametric-COVID-19, (accessed on 18 March 2021).

## Abbreviations

The following syntax and abbreviations are used in this manuscript:

AFCR	Asymptotic Fatality–Case Ratio
Generation time	Average time between two consecutive infections
IFCR	Instantaneous Fatality–Case Ratio
$R_{0}$	Basic Reproduction Number, number of secondary infections emerging
	from an index case in a fully susceptible population
Serial interval	Average time between the onset of symptoms of two consecutive
	infections, often used as an approximation to the generation time
SIR/SEIR	Susceptible-(exposed)-infected-removed epidemiological
	compartment models
Time-to-death duration	Average time between the registrations of new cases and the
	corresponding registrations of deaths, if applicable.
